# Modelling time-varying genetic effects on binary disease risk via functional Mendelian randomization

**DOI:** 10.1093/bioinformatics/btag442

**Published:** 2026-08-21

**Authors:** Nicole Fontana, Piercesare Secchi, Emanuele Di Angelantonio, Francesca Ieva

**Affiliations:** MOX, Department of Mathematics, Politecnico di Milano, Milan, 20133, Italy; Health Data Science Centre, Human Technopole, Milan, 20157, Italy; MOX, Department of Mathematics, Politecnico di Milano, Milan, 20133, Italy; Health Data Science Centre, Human Technopole, Milan, 20157, Italy; BHF Cardiovascular Epidemiology Unit, Department of Public Health and Primary Care, University of Cambridge, Cambridge, CB2 0BB, United Kingdom; Victor Phillip Dahdaleh Heart and Lung Research Institute, University of Cambridge, Cambridge, CB2 0BB, United Kingdom; BHF Centre of Research Excellence, University of Cambridge, Cambridge, CB2 0BB, United Kingdom; NIHR Blood and Transplant Research Unit in Donor Health and Behaviour, University of Cambridge, Cambridge, United Kingdom; MOX, Department of Mathematics, Politecnico di Milano, Milan, 20133, Italy; Health Data Science Centre, Human Technopole, Milan, 20157, Italy

## Abstract

**Motivation:**

Genome-wide association studies have identified thousands of genetic variants associated with complex traits, establishing Mendelian randomization (MR) as a powerful framework for causal inference using variants as natural experiments. However, existing MR methods treat causal effects as static, relying on cross-sectional exposure measurements and ignoring how genetic predispositions to disease operate dynamically across the life course. Recovering age-specific causal effect functions from longitudinal data requires combining functional data representations of exposure trajectories with instrumental variable estimation strategies suitable for binary disease endpoints, a methodological gap that has remained unaddressed.

**Results:**

We develop a functional MR framework for binary outcomes that integrates functional principal component analysis with two-stage residual inclusion (2SRI), ensuring consistent estimation under the nonlinear logistic link function that renders standard instrumental variable estimators inconsistent. Simulations across different causal effect trajectory shapes, varying measurement densities, and varying instrument strengths demonstrate accurate recovery of time-varying genetically predicted effects with minimal bias. Applied to UK Biobank data, the framework identifies an age-specific causal effect of genetically predicted body mass index on type 2 diabetes risk concentrated in early mid-adulthood and progressively attenuating thereafter. Concordance between the proposed 2SRI estimator applied to type 2 diabetes and the established continuous-outcome functional MR estimator applied to the paired glycated haemoglobin marker in the same cohort provides indirect empirical support for the validity of the proposed approach.

**Availability and implementation:**

The method is implemented in the R package mvfmr, with a full tutorial vignette.

## 1. Introduction

Population genomics studies, from genome-wide association studies (GWAS) to large biobank-scale cohorts, have identified thousands of genetic variants associated with complex traits and disease outcomes. These variants serve as powerful instruments for studying genetic predispositions to these phenotypes via Mendelian randomization (MR). MR uses genetic variants associated with an exposure as instrumental variables to estimate the causal effect of that exposure on a clinical outcome. By exploiting the random inheritance of alleles at conception as a ‘natural experiment’, MR helps address endogeneity arising from unmeasured confounding and reverse causality that affect observational analyses (Sanderson *et al.* 2022). However, a fundamental limitation of existing MR approaches is that they treat effects as static and time-averaged, relying on exposure measurements obtained at a single time point (Labrecque and Swanson 2020). This assumption is biologically implausible for many complex traits that evolve continuously across the life course. The effects of exposure are therefore inherently dynamic, potentially intensifying or diminishing over time and creating age-specific periods of elevated effect in which timing can be as decisive as magnitude (Wagner *et al.* 2021). Characterizing these temporal patterns is essential for identifying the age-specific periods during which genetic predispositions exert their strongest biological influence. Accordingly, modelling longitudinal exposures as trajectories, rather than scalar summaries, is crucial for capturing the time-varying structure of genetically mediated effects (Wagner *et al.* 2024), an approach supported by empirical evidence that genetic associations with complex traits vary in magnitude across developmental stages (Winkler *et al.* 2015, 2024).

Recent methodological advances have introduced functional MR frameworks based on functional principal component analysis (FPCA) to model longitudinal exposure trajectories as smooth functions of age, enabling estimation of a continuously time-varying causal effect function (Tian *et al.* 2024). FPCA extracts the main patterns of variation from the observed trajectories, representing each individual’s trajectory as a low-dimensional combination of these patterns while preserving the key temporal features of the data. This approach represents a significant step beyond static GWAS-based MR; however, it has been validated exclusively for continuous outcomes. Many primary endpoints in genetic epidemiology are binary, including disease incidence, mortality, and treatment response. Extending functional MR to models with binary outcomes and therefore nonlinear link functions presents a fundamental methodological challenge: the nonlinearity of the logistic link function renders standard instrumental variable estimators, such as two-stage predictor substitution, statistically inconsistent (Terza *et al.* 2008, Liu *et al.* 2010). An estimation strategy is therefore needed that accommodates this nonlinearity while preserving the functional representation of the exposure trajectory. Moreover, existing life-course MR studies rely on coarse comparisons between broad developmental stages or aggregate cumulative measures (Cao *et al.* 2016, Richardson *et al.* 2020); the framework proposed here instead recovers a continuous age-specific causal effect function, providing higher temporal resolution for characterizing genetic predispositions.

In this work, we propose a functional MR framework for binary outcomes by integrating FPCA with two-stage residual inclusion (2SRI). Unlike traditional substitution methods, 2SRI ensures consistent estimation even with nonlinear link functions, providing a robust solution to endogeneity in binary outcomes (Terza *et al.* 2008, Wooldridge 2015). 2SRI augments the outcome model with the residuals from the first-stage regression, which directly adjust the endogenous variation not explained by the instruments. We validate the framework through extensive simulations across four causal effect trajectory scenarios and assess its robustness to measurement sparsity and weak instrument strength. We apply the method to estimate the genetically predicted time-varying causal effect of body mass index (BMI) on type 2 diabetes (T2D) risk using longitudinal data from the UK Biobank, leveraging 199 GWAS-derived BMI instruments. Then, since direct benchmarking against existing functional MR methods is not feasible, as the only available approach (Tian *et al.* 2024) is restricted to continuous outcomes, we exploit the biological relationship between T2D and glycated haemoglobin (HbA1c) as an indirect empirical comparison. Specifically, by applying the continuous-outcome estimator to HbA1c and the proposed 2SRI estimator to T2D in the same cohort, we obtain a paired evaluation of two methodologically distinct estimators on the same underlying glycaemic phenomenon.

This paper makes three contributions. First, methodologically, we provide a functional MR framework for binary endpoints via 2SRI, applicable to any time-varying exposure with valid GWAS-derived genetic instruments. Second, computationally, we release an open-source implementation in the R package mvfmr (CRAN), enabling immediate application to any longitudinal cohort study working both with individual-level and summary-level data. Third, empirically, we demonstrate how genetic predispositions to adiposity exert age-specific effects on disease risk, providing a temporal characterization that moves beyond static, time-averaged MR estimates.

## 2. Materials and methods

### 2.1 Model and estimation strategy

#### 2.1.1 Functional causal model formulation

**Figure 1:**
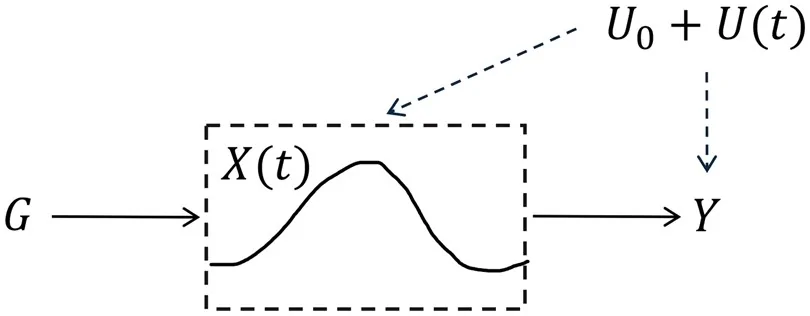
Causal structure. Genetic variants G serve as instruments for the time-varying exposure X(t), which affects the binary outcome *Y*. Unmeasured confounders $U_{0}+U(t)$ affect both the exposure trajectory and outcome.

**Figure 2:**
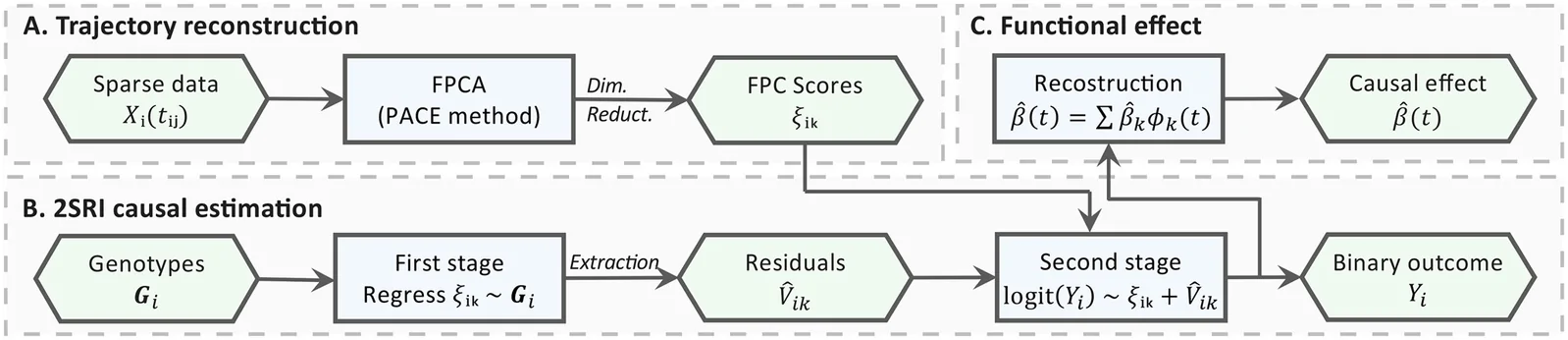
Workflow of the proposed functional Mendelian randomization framework for binary outcomes. (A) Sparse, irregular longitudinal measurements are decomposed via FPCA (PACE method) to extract eigenfunctions $\phi_{k}(t)$ and individual scores $\xi_{ik}$, with the scores used as low-dimensional representations of the longitudinal exposure trajectories. (B) Endogeneity is addressed via a two-stage residual inclusion (2SRI) approach to estimate the causal coefficients ${\hat{\beta }}_{k}$, representing the effects of the scores on the outcome. (C) The estimated coefficients ${\hat{\beta }}_{k}$ are projected onto the eigenfunctions to reconstruct the time-varying causal effect function $\hat{\beta }(t)$, with pointwise 95% confidence intervals via non-parametric bootstrap.

Given a cohort of individuals i=1,…,n for whom we observe a time-varying exposure $X_{i}(t)$ measured over period $t\in [t_{0},t_{1}]$, a binary outcome $Y_{i}\in \{0,1\}$, and genetic data comprising *L* single nucleotide polymorphisms (SNPs) denoted $G_{i}={\left(G_{i1},\ldots ,G_{iL}\right)}^{⊤}$. Our primary objective is to estimate the genetically time-varying causal effect function β(t), which quantifies the age-specific impact of the exposure on disease risk. As illustrated in the causal structure in Fig. 1, we assume the genetic instruments $G_{i}$ influence the outcome $Y_{i}$ exclusively through the exposure trajectory $X_{i}(t)$. We model the causal relationship between the longitudinal exposure and the binary outcome through a functional logistic regression model:


(1) $$\text{logit}[P(Y_{i}=1)]=\beta_{0}+\int_{t_{0}}^{t_{1}}\beta (t)X_{i}(t)dt+g_{Y}(U_{i}),$$

where β(t) represents the change in log-odds of the outcome per unit increase in exposure at age *t*. The integral term captures the cumulative causal effect, weighting the exposure trajectory by its time-varying impact across the life course. The term $g_{Y}(U_{i})$ accounts for unmeasured confounders, potentially encompassing both time-invariant ($U_{i0}$) and time-varying ($U_{i}(t)$) factors, which, under MR assumptions, are assumed to be independent of the genetic instruments $G_{i}$. The full estimation workflow is shown in Fig. 2.

#### 2.1.2 FPCA for the longitudinal exposure

To represent the longitudinal exposure as a smooth function of age rather than a set of discrete repeated measurements, we apply FPCA (Fig. 2A). The functional logistic regression in Equation (1) is inherently infinite-dimensional, making direct estimation of β(t) infeasible. FPCA addresses this by projecting exposure trajectories into a finite-dimensional space. Unlike fixed bases (e.g. B-splines), FPCA provides an optimal, data-driven representation by decomposing trajectories into a small set of orthogonal eigenfunctions that capture the dominant modes of variation while preserving the key features of individual trajectories. For the sparse and irregularly sampled longitudinal data typical in healthcare settings, we implement FPCA using the PACE (principal analysis by conditional estimation) method (Yao *et al.* 2005). PACE first estimates the mean function μ(t) via local polynomial smoothing of all available measurements pooled across individuals. It then estimates the covariance surface G(s,t)=Cov[X(s),X(t)], from which eigenfunctions $\phi_{k}(t)$ are obtained as solutions to the eigenequation $\int G(s,t)\phi_{k}(s)ds=\lambda_{k}\phi_{k}(t)$. Individual FPC scores $\xi_{ik}$ are predicted via conditional expectation, effectively ‘borrowing strength’ from the entire cohort to reconstruct trajectories for individuals with limited observations. The exposure trajectories are thus approximated as:


(2) $$X_{i}(t)\approx \mu (t)+\sum_{k=1}^{K}\xi_{ik}\phi_{k}(t).$$

The scores $\xi_{ik}$ summarize how an individual’s trajectory deviates from the average and serve as the low-dimensional representation of the exposure trajectories in our causal model. Crucially, since the original trajectories $X_{i}(t)$ are subject to unmeasured confounding, the FPC scores inherit this endogeneity and serve as the endogenous predictors in our instrumental variable framework. We select the truncation parameter *K* by maximizing the area under the ROC curve (AUC) for the final outcome prediction within a cross-validation framework. Specifically, FPCA models with different numbers of retained components are fitted, and the value of *K* yielding the best predictive performance on held-out data is selected. This supervised selection ensures that the retained components prioritize temporal variations most relevant to the disease aetiology while acting as a regularization step against high-frequency noise.

#### 2.1.3 Instrumental variable estimation via 2SRI

To obtain a consistent estimate of β(t) in the presence of unmeasured confounding, we adopt an MR framework using genetic variants as instrumental variables. MR relies on the three core MR assumptions:

- Relevance: the genetic instruments must be associated with the exposure trajectory, throughout the period of interest.
- Independence: the genetic instruments must be independent of the confounders that affect both the exposure and the outcome.
- Exclusion restriction: the instruments affect the outcome only through their impact on the exposure trajectory.

The relevance assumption is verified through conditional F-statistics for each FPC. The independence assumption is supported by the random segregation of alleles at conception. While the exclusion restriction is untestable, using multiple genome-wide significant variants provides protection against violations from pleiotropy. In addition, the validity of life-course MR relies on the hypothesis that the genetic architecture of biological traits is dynamic, exhibiting age-dependent associations over the life course.

Given the binary nature of our outcome, we implement the 2SRI estimator (Terza *et al.* 2008, Wooldridge 2015). In non-linear models, like logistic regression, the conventional two-stage predictor substitution (2SPS) approach, where the endogenous variable is replaced by its predicted value from the first stage, leads to inconsistent estimates. The inconsistency arises because the nonlinear logistic link function invalidates the substitution of predicted values, thereby distorting the conditional mean structure (Cai *et al.* 2011, Zhang and Lewsey 2024). 2SRI overcomes this by including the first-stage residuals as additional covariates in the second-stage model, effectively acting as a control function for the unmeasured confounding (Fig. 2B). In the first stage, we regress each of the *K* endogenous FPC scores on the genetic instruments to isolate the genetically-driven component of the trajectory:


(3) $$\xi_{ik}=\alpha_{0k}+G_{i}^{⊤}\alpha_{k}+\epsilon_{ik},\;{\hat{V}}_{ik}=\xi_{ik}-{\hat{\xi }}_{ik},$$

where ${\hat{V}}_{ik}$ represents the residuals capturing the variation in FPC scores not explained by the instruments (i.e. potential confounding). In the second stage, we include both the original FPC scores and the first-stage residuals in a logistic regression:


(4) $$\text{logit}[P(Y_{i}=1)]=\beta_{0}+\sum_{k=1}^{K}\beta_{k}^{*}\xi_{ik}+\sum_{k=1}^{K}\delta_{k}{\hat{V}}_{ik},$$

where the coefficients $\beta^{*}={\left(\beta_{1}^{*},\ldots ,\beta_{K}^{*}\right)}^{⊤}$ estimate the causal effect of the FPC scores on the outcome, while $\delta ={\left(\delta_{1},\ldots ,\delta_{K}\right)}^{⊤}$ captures the correlation between FPC scores and unobserved confounders, correcting for endogeneity. Finally, the time-varying causal effect function is recovered (Fig. 2C), by projecting the estimated coefficients back onto the functional basis:


(5) $$\hat{\beta }(t)=\sum_{k=1}^{K}{\hat{\beta }}_{k}^{*}\phi_{k}(t).$$

We employ a nonparametric bootstrap procedure to compute confidence intervals. By resampling individuals with replacement, we construct 95% pointwise confidence intervals for $\hat{\beta }(t)$ using the $2.5^{\text{th}}$ and $97.5^{\text{th}}$ percentiles of the bootstrap distribution at each time point *t*. This procedure ensures that our inference accounts for the uncertainty propagated through all stages of the functional IV estimation process.

### 2.2 Simulation design

We evaluated the performance of the proposed method using 500 independent replications per scenario, with a sample size of N=5000 individuals and J=30 independent genetic variants as instrumental variables. Genotypes were generated as $G_{ij}∼\text{Binomial}(2,0.3)$, assuming the minor allele frequency is 0.3. Individual exposure trajectories $X_{i}(t)$ were simulated over t∈[0,50] according to:


(6) $$X_{i}(t)=\sum_{j=1}^{J}G_{ij}\gamma_{j}(t)+[U_{i0}+U_{i}(t)]+\epsilon_{i}(t),$$

where the genetic effects $\gamma_{j}(t)$ combine linear and quadratic trends to allow for age-dependent genetic architectures. The residual term $\varepsilon_{j}(t)$ accounts for exposure-specific noise and measurement error. The latent process $U_{j}(t)$ captures time-varying unobserved confounding, while $U_{0j}∼N(0,1)$ represents baseline unobserved heterogeneity. Both $U_{j}(t)$ and the residual term $\varepsilon_{j}(t)$ are modelled as discretized Wiener processes. To mimic the sparsity of real-world longitudinal cohorts, we sampled $n_{\text{sparse}}$ observations per individual at random time points within the window. Binary outcomes were generated using a functional logistic regression model:


(7) $$\text{logit}[P(Y_{i}=1)]=\beta_{0}+\int_{0}^{50}\beta (t)X_{i}(t)dt+g_{Y}(U_{i}),$$

where β(t) denotes the genetically predicted time-varying causal effect function, and $g_{Y}(U_{i})$ captures the contribution of unmeasured confounding to the outcome. We considered four epidemiologically plausible patterns for the causal effect β(t):

- **Constant** (β(t)=0.1): time-invariant baseline.
- **Early life** (β(t)=0.1·I(t<20)): early life effect.
- **Linear** (β(t)=0.02·t): progressive risk accumulation.
- **Quadratic** ($\beta (t)=0.002\cdot t^{2}-0.11\cdot t+0.5$): U-shaped age dependence.

Performance was evaluated using the mean integrated squared error (MISE), which quantifies the overall accuracy of the estimated time-varying causal effect function, and pointwise coverage, defined as the proportion of time points at which the true effect function lies within the corresponding 95% pointwise bootstrap confidence interval. Instrument strength was monitored via marginal (*FF*) and conditional F-statistics (*cFF*) for each principal component.

### 2.3 UK Biobank data

We used data from the UK Biobank, a prospective cohort of approximately 500 000 participants recruited between 2006 and 2010 in the UK (Sudlow *et al.* 2015). To minimize genetic confounding, we restricted the analysis to individuals of European ancestry (*N* = 442 434). After excluding participants with incomplete genetic data or prevalent T2D at age 50, 418 207 individuals remained eligible. Incident T2D was defined via hospital admission records (ICD-10 code E11) and primary care diagnoses, with follow-up continuing until diagnosis, death, or administrative censoring in May 2022. Longitudinal BMI measurements were extracted from assessment centre visits and primary care records, focusing on the age range of 50 to 70 years and considering only measurements recorded prior to T2D diagnosis. Genetic instruments for BMI were derived from 199 independent SNPs identified in a large-scale GWAS from the GWAS Catalog study GCST005951 (Winkler *et al.* 2015), conducted in a European population. The validity of using longitudinal BMI trajectories as a time-varying exposure in the MR framework is supported by evidence that genetic associations with BMI vary in magnitude across the life course (Winkler *et al.* 2015 2024), confirming that the 199 GWAS-derived instruments capture age-dependent genetic effects on adiposity rather than a static phenotype.

To ensure robustness against different longitudinal sampling densities, we defined two distinct analytic groups. The *main cohort* comprises 1 11 360 participants with at least three BMI measurements between ages 50 and 70, providing a good balance between sample size and trajectory reconstruction quality. The *sensitivity cohort* of 12 027 participants with at least 10 BMI observations was used to verify the consistency of the estimated effects when trajectories are captured with higher precision. Baseline characteristics, presented in Table 1, available as supplementary data at *Bioinformatics* online, show that the sensitivity cohort exhibits a higher burden of comorbidities and medication use, likely reflecting more frequent healthcare interactions.

**Table 1:** Performance of functional MR across different effect shapes.^a^

Effect pattern	MISE	Coverage
Constant	0.0003 (0.0003)	0.953 (0.115)
Early life effect	0.0009 (0.0002)	0.453 (0.078)
Linear	0.0431 (0.0107)	0.159 (0.065)
Quadratic	0.1159 (0.0922)	0.580 (0.153)

a Values shown as mean (SD) over 500 simulation replications.

As a method comparison and biological validation, we used HbA1c as a continuous marker of glycaemic control, with data sourced from primary care records. Since T2D and HbA1c measure the same underlying glycaemic phenotype via different outcome types, this analysis enables a direct empirical comparison between the proposed 2SRI estimator for binary outcomes and the standard functional MR estimator for continuous outcomes (Tian *et al.* 2024). Sample sizes for the HbA1c analyses (*N* = 105 551 for the main and *N* = 11 475 for the sensitivity analysis) are slightly reduced compared to the T2D analysis due to the more limited availability of blood biochemistry data; a detailed description of this cohort is available in Table 2, available as supplementary data at *Bioinformatics* online.

**Table 2:** Performance of functional MR with varying number of sparse observations per subject.^a^

Observations per subject	MISE	Coverage
5	0.1305 (0.0380)	0.070 (0.049)
10	0.0431 (0.0107)	0.159 (0.065)
20	0.0067 (0.0047)	0.689 (0.252)
35	0.0048 (0.0054)	0.897 (0.183)
50	0.0040 (0.0037)	0.902 (0.147)

a Values shown as mean (SD) over 500 simulation replications.

### 2.4 Computational implementation

The proposed framework is implemented in the mvfmr R package, available on CRAN (Fontana *et al.* 2026). The package provides a unified environment for functional MR analyses, including both univariable functional MR for continuous outcomes as introduced by Tian *et al.* (2024) and the binary-outcome extension based on 2SRI proposed in this paper. The proposed framework is implemented using the function fmvmr_separate, which requires three inputs: (i) individual-level longitudinal exposure measurements, which may be sparse and irregularly observed; (ii) a binary outcome vector; and (iii) a matrix of SNP genotypes used as genetic instruments. For settings where individual-level genetic data are unavailable, the function also supports summary-level genetic data inputs for two-sample MR. The package internally performs the FPCA step using the PACE method, via the fdapace package (Zhou *et al.* 2024), and automatically selects the number of functional principal components *K* through cross-validation based on AUC maximization. In addition to the 2SRI estimator presented in this work, the package also implements a 2SRI-LASSO estimator, which combines the 2SRI framework with LASSO regularization and may improve stability in settings with many correlated genetic instruments. The main output of the function is the estimated time-varying causal effect function $\hat{\beta }(t)$ evaluated on a user-specified time grid, together with pointwise 95% bootstrap confidence intervals. The package also provides diagnostic outputs to assess model adequacy and instrument strength, including conditional F-statistics for each functional principal component, first-stage residuals, FPCA eigenfunction plots, and performance metrics such as mean integrated squared error (MISE) and coverage. Parallel computation is supported to improve computational efficiency. A comprehensive tutorial vignette is included in the package documentation, illustrating the complete workflow for univariable functional MR, from data preparation and FPCA diagnostics to 2SRI estimation and visualization of the estimated causal effect function. The vignette provides a reproducible simulated example and serves as a practical guide for applied studies.

## 3. Results

### 3.1 Simulation results

We first evaluated the method’s ability to recover various functional forms of the time-varying causal effect with a fixed measurement density of $n_{\text{sparse}}=10$. As summarized in Table 1, the framework achieved low MISE across all considered effect structures. As the complexity of the underlying effect increased, we observed a gradual increase in the MISE, which remains within an acceptable range for reliable interpretation. While the pointwise coverage for complex patterns was lower than the nominal 95%, the point estimates remained highly accurate and successfully tracked the true functional shape. The decrease in coverage suggests that while the point estimates are accurate, the pointwise bootstrap confidence intervals tend to be overly overconfident (narrow). This behaviour likely reflects the challenge of propagating uncertainty from the FPCA stage, where trajectories are reconstructed from sparse data, into the non-linear 2SRI estimator. Instrument strength for all scenarios is shown in Table 3, available as supplementary data at *Bioinformatics* online.

**Table 3:** Performance under varying instrument strength configurations.^a^

Scenario	cFF (PC1)	cFF (PC2)	MISE
Both moderate-weak	5.67 (0.74)	4.64 (0.51)	0.0610 (0.0107)
PC1 moderate, PC2 weak	7.04 (0.90)	4.57 (0.52)	0.0613 (0.0109)
PC1 strong, PC2 weak	10.21 (1.29)	5.21 (0.59)	0.0595 (0.0095)
Both weak	3.81 (0.52)	3.21 (0.36)	0.0603 (0.0103)

a cFF for first and second principal components varied through differential scaling of genetic effects. Values shown as mean (SD) over 500 simulation replications.

Then, we assessed the impact of measurement frequency by varying $n_{\text{sparse}}\in \{5,10,20,35,50\}$ for a linear effect. This sensitivity analysis reflects the trade-off between data collection burden and estimation accuracy common in practice. Results in Table 2 revealed a clear positive correlation between data density and estimation performance. Notably, the method maintained reasonable accuracy even with limited observations, demonstrating its robustness for applications in longitudinal cohorts with irregular sampling. Instrument strength remained consistent across measurement densities, as reported in Table 4, available as supplementary data at *Bioinformatics* online.

Finally, to assess robustness to weak instrument bias, we evaluated performance across varying instrument strength configurations by differentially scaling genetic effects on trajectory intercept and temporal trend components in Equation (6). We examined four scenarios as shown in Table 3: (i) both components moderately weak; (ii) first component moderate with weak second component; (iii) first component strong with weak second component; and (iv) both components weak. Across all scenarios, including those with cFF<5, the MISE remained remarkably stable.

### 3.2 Genetically predicted time-varying causal effect of BMI on T2D

**Figure 3:**
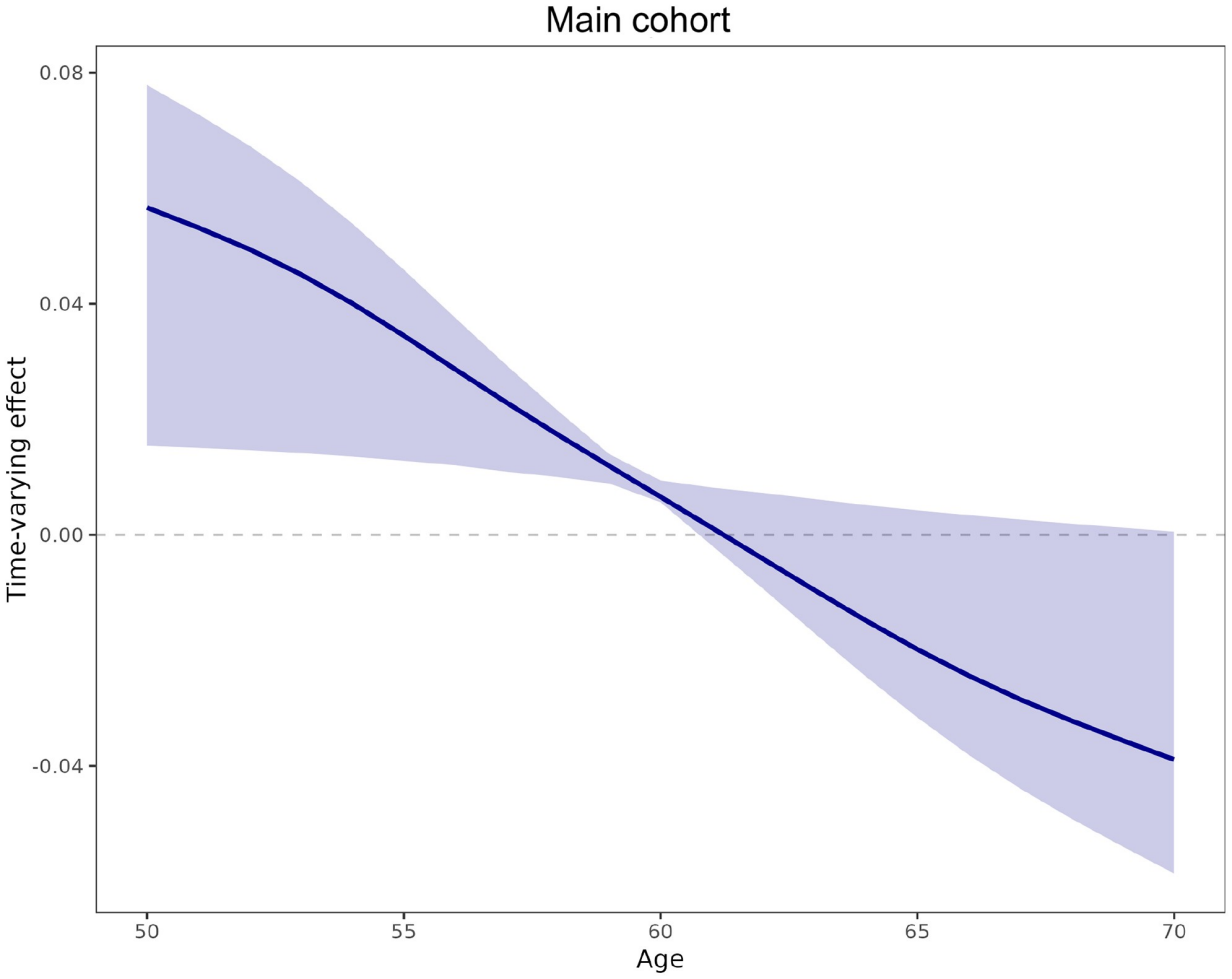
Genetically predicted time-varying causal effect of BMI on T2D risk in the main cohort. The line represents point estimates of $\hat{\beta }(t)$ (change in log-odds of T2D per SD increase in BMI at age *t*), with shaded regions indicating pointwise 95% confidence intervals based on 100 bootstrap samples. The dashed horizontal line at zero indicates no effect.

In the main cohort, 9346 participants (8.4%) developed incident T2D. FPCA identified two principal components, shown in Fig. 1, available as supplementary data at *Bioinformatics* online, explaining 98.89% of the variance in BMI trajectories (FPC1: 90.22%; FPC2: 8.67%). The first eigenfunction $\phi_{1}(t)$ represented overall BMI level throughout the 20-year window, while the second $\phi_{2}(t)$ captured linear changes (rising or falling trends) over time. Instrument relevance was confirmed by a conditional F-statistic of $F_{1}=16.11$ for the first component, significantly exceeding the conventional threshold for strong instruments. The second component showed a lower association ($F_{2}=2.21$); this is biologically expected, as genetic variants identified in large-scale cross-sectional GWAS primarily capture stable phenotypic differences rather than the more nuanced within-individual temporal dynamics represented by FPC2. The estimated causal effect function $\hat{\beta }(t)$ shown in Fig. 3 revealed that the impact of genetically predicted BMI on T2D risk is highly age-dependent. The effect is strongest and statistically significant at age 50, remaining positive through mid-adulthood. However, we identified a marked attenuation of risk beginning in the early 60 s. Specifically, after this age, the 95% confidence intervals contain zero, indicating that the genetically predicted causal effect of BMI on T2D risk is no longer statistically significant in this later life stage. These findings provide a more complete picture than conventional static MR. In the existing literature, time-averaged MR estimates report an overall odds ratio of 2.80 (95% CI: 1.89–4.15) (Richardson *et al.* 2020). However, our functional approach demonstrates that these static measures mask important temporal heterogeneity: the causal effect of BMI is not a lifelong constant but is predominantly concentrated in early mid-adulthood, followed by a significant attenuation as individuals transition into their 60 s.

To ensure these results were not driven by measurement frequency or irregular sampling, we replicated the analysis in the sensitivity cohort. Despite the smaller sample size and a higher incidence of T2D (33.3%), the estimated causal function remained broadly consistent with the main analysis, showing a positive and significant BMI effect during the 50 s and early 60 s, followed by attenuation towards non-significance at older ages (Figs 2 and 3, available as supplementary data at *Bioinformatics* online). Although effect magnitudes were smaller in the sensitivity cohort, likely due to stricter inclusion criteria and reduced statistical power, the concordant temporal pattern across cohorts suggests that the diminishing genetically predicted effect of BMI in late adulthood is a robust finding rather than a methodological artefact.

### 3.3 Method comparison: 2SRI estimator versus functional MR on a continuous glycaemic marker

HbA1c, a continuous marker of glycaemic control, quantifies the same underlying glycaemic dysregulation that T2D captures as a binary clinical event. This makes it uniquely suited for a paired methodological comparison: by applying the proposed 2SRI-based estimator to T2D diagnosis and the standard continuous-outcome functional MR estimator (Tian *et al.* 2024) to HbA1c, we can directly assess whether the two approaches recover concordant causal effect functions when applied to distinct definitions of the same biological process. Cross-validation selected 2 functional principal components for the HbA1c analysis (Figs 4 and 5, available as supplementary data at *Bioinformatics* online). The estimated causal effect of BMI on HbA1c (Figs 6 and 7, available as supplementary data at *Bioinformatics* online) shows a clear temporal pattern: positive and statistically significant from age 50 until approximately age 60, followed by progressive attenuation towards non-significance in later years. The temporal concordance between the T2D (binary, 2SRI estimator) and HbA1c (continuous, standard functional MR estimator) provides empirical support for the validity of the 2SRI approach in the binary-outcome setting, since the two outcomes are two representations of the same underlying glycaemic information, one discrete, one continuous, processed through methodologically distinct estimators.

## 4. Discussion

We have developed and validated a univariable functional MR framework for binary outcomes, addressing a key methodological gap in causal inference for time-varying exposures. By combining FPCA with 2SRI estimation, our approach identifies age-specific causal effects while properly accounting for the non-linear relationship between longitudinal trajectories and binary disease outcomes. Unlike traditional MR, which provides a static average effect, functional MR recovers the dynamic evolution of genetically mediated risk over time, a temporal characterization that is essential for identifying age-specific periods of elevated effect. Simulation studies confirmed the method’s ability to recover diverse time-varying effect patterns across varying levels of measurement sparsity and genetic instrument strength, demonstrating high precision with minimal bias and establishing the robustness of the approach under varying degrees of effect complexity.

Applied to UK Biobank data, the framework reveals that the genetically predicted causal effect of BMI on T2D risk is concentrated in the early fifties and progressively attenuates, becoming statistically negligible in later adulthood. This declining pattern is consistent with the accumulation of causal effect over the exposure trajectory in earlier decades, after which the marginal contribution of contemporaneous BMI elevation to disease risk diminishes. Crucially, the striking concordance between the T2D and HbA1c results demonstrates that both estimators recover the same age-specific causal effect structure, providing empirical evidence that the proposed 2SRI approach for binary outcomes agrees with the established continuous-outcome functional MR method when applied to paired outcomes measuring the same underlying glycaemic process.

Several methodological limitations should be noted. First, while the first FPC exhibited strong instrument strength, the second component showed weaker genetic associations, a general challenge in functional IV models where higher-order eigenfunctions capture more complex trajectory dynamics that are harder to instrument from cross-sectional GWAS. Although our simulations confirmed that 2SRI maintains low bias even under these conditions, caution is warranted when interpreting finer nuances of trajectory slope effects. Second, horizontal pleiotropy remains a potential concern in any MR application; functional MR-Egger and multivariable functional MR (Fontana *et al.* 2025) represent the natural next step for pleiotropy-robust functional causal inference. Third, our analysis was restricted to participants of European ancestry within the UK Biobank; replication in diverse ancestral cohorts is essential to assess the generalizability of the estimated age-varying effects.

The functional MR framework developed here is broadly applicable beyond the proposed context: it can be applied to any time-varying exposure measured in a longitudinal cohort with available GWAS-derived instruments or summary-level genetic data. The proposed method is available in the R package mvfmr, enabling immediate application without requiring manual implementation of the FPCA or 2SRI steps. Future algorithmic directions include pleiotropy-robust functional estimators and extensions to time-to-event outcomes. More broadly, the framework provides a new computational lens for population genomics studies seeking to move beyond static GWAS associations towards a dynamic characterization of how genetic predispositions unfold across the life course.

## Acknowledgements

This work was supported by MUR, grant Dipartimento di Eccellenza 2023–2027. F.I. acknowledges the National Plan for NRRP Complementary Investments ‘Advanced Technologies for Human-centred Medicine’ (PNC0000003). Large language models were used as an aid to correct written text and as an aid for code writing.

## Supplementary Material

btag442_Supplementary_Data

## Data Availability

This research has been conducted using the UK Biobank Resource under Application Number 102297. UK Biobank data are accessible for health-related research in the public interest. Code and tutorial vignette are available at https://CRAN.R-project.org/package=mvfmr.
