# Supplementary material for: Modelling time-varying genetic effects on binary disease risk via functional Mendelian randomization
Source: Bioinformatics. 2026 Aug 21;42(Suppl 2):btag442. doi: 10.1093/bioinformatics/btag442 (PMC13501298; doi:10.1093/bioinformatics/btag442)
Supplement: Supplementary file 1 [file btag442_supplementary_data.pdf]

Supplementary Material

Modeling time-varying genetic effects on binary disease risk via functional Mendelian Randomization

Nicole Fontana[1,2], Piercesare Secchi[1], Emanuele Di Angelantonio[2,3,4,5,6], Francesca Ieva[1,2]

[1] MOX, Department of Mathematics, Politecnico di Milano, Milan, Italy
[2] Health Data Science Research Centre, Human Technopole, Milan, Italy
[3] BHF Cardiovascular Epidemiology Unit, University of Cambridge, Cambridge, UK
[4] Victor Phillip Dahdaleh Heart and Lung Research Institute, University of Cambridge, UK
[5] BHF Centre of Research Excellence, University of Cambridge, UK
[6] NIHR Blood and Transplant Research Unit in Donor Health and Behaviour, University of Cambridge, UK

Supplementary Tables

Supplementary Table S1

Table S1 Baseline characteristics of study participants in the primary analysis (BMI on T2D), stratified by cohort.

**Characteristic**	**Main cohort ($N = 111{,}360$)**	**Sensitivity cohort ($N = 12{,}027$)**
Female sex, n (%)	50,504 (45.4%)	6,826 (52.0%)
Current smoker, n (%)	10,346 (9.3%)	1,173 (8.9%)
**Clinical history**		
Atrial fibrillation, n (%)	493 (0.4%)	95 (0.7%)
Heart failure, n (%)	867 (0.8%)	117 (0.9%)
Ischemic heart disease, n (%)	1,982 (1.8%)	749 (5.7%)
Atherosclerotic cardiovascular disease, n (%)	884 (0.8%)	107 (0.8%)
Stroke, n (%)	679 (0.6%)	147 (1.1%)
Hypertension, n (%)	12,140 (10.9%)	2,814 (21.4%)
Type 1 diabetes, n (%)	259 (0.2%)	162 (1.2%)
**Follow-up and outcomes**		
Incident Type 2 diabetes cases, n (%)	9,346 (8.39%)	4,010 (33.34%)

Supplementary Table S2

Table S2 Baseline characteristics of study participants in the secondary analysis (BMI on HbA1c), stratified by cohort.

**Characteristic**	**Main cohort ($N = 105{,}551$)**	**Sensitivity cohort ($N = 11{,}475$)**
Female sex, n (%)	47,764 (45.3%)	5,855 (51.0%)
Current smoker, n (%)	9,764 (9.3%)	1,021 (8.9%)
**Clinical history**		
Atrial fibrillation, n (%)	452 (0.4%)	81 (0.7%)
Heart failure, n (%)	814 (0.8%)	103 (0.9%)
Ischemic heart disease, n (%)	1,848 (1.8%)	616 (5.4%)
Atherosclerotic cardiovascular disease, n (%)	840 (0.8%)	94 (0.8%)
Stroke, n (%)	637 (0.6%)	127 (1.1%)
Hypertension, n (%)	11,367 (10.8%)	2,374 (20.7%)
Type 1 diabetes, n (%)	231 (0.2%)	121 (1.1%)
Gestational diabetes, n (%)	86 (0.1%)	12 (0.1%)
**Follow-up and outcomes**		
Incident Type 2 diabetes cases, n (%)	8,426 (7.98%)	3,825 (33.33%)

Supplementary Table S3

Table S3 F-statistics for instrument strength across effect shapes. Values shown as mean (SD) over 500 simulation replications.

Effect	Standard F-statistic (FF)			Conditional F-statistic (cFF)		
**Pattern**	**PC1**	**PC2**	**PC3**	**PC1**	**PC2**	**PC3**
Constant	9.26 (2.12)	8.47 (1.96)	—	8.46 (2.01)	7.76 (1.96)	—
Early life	9.26 (2.12)	8.47 (1.96)	1.43 (0.48)	8.41 (2.04)	7.71 (1.98)	1.22 (0.40)
Linear	9.26 (2.12)	8.47 (1.96)	—	8.46 (2.01)	7.76 (1.96)	—
Quadratic	9.26 (2.12)	8.47 (1.96)	1.43 (0.36)	7.31 (1.88)	6.61 (1.97)	1.12 (0.29)

FF = standard F-statistic testing association between instruments and each principal component; cFF = conditional F-statistic accounting for other principal components. "—" indicates that cross-validation selected $K = 2$ components (no PC3 was retained). Values shown as mean (SD) over 500 simulation replications.

Supplementary Table S4

Table S4 F-statistics for instrument strength with varying numbers of observations per subject (linear effect). Values shown as mean (SD) over 500 simulation replications.

Obs. per	Standard F-statistic (FF)			Conditional F-statistic (cFF)		
**subject**	**PC1**	**PC2**	**PC3**	**PC1**	**PC2**	**PC3**
5	8.87 (2.23)	6.92 (1.96)	0.78 (0.23)	8.20 (2.14)	6.42 (1.92)	0.79 (0.21)
10	9.26 (2.12)	8.47 (1.96)	—	8.46 (2.01)	7.76 (1.96)	—
20	9.34 (2.15)	9.16 (2.12)	—	8.50 (2.01)	8.37 (2.13)	—
35	9.36 (2.15)	9.35 (2.12)	—	8.52 (2.03)	8.54 (2.14)	—
50	9.37 (2.15)	9.43 (2.13)	—	8.51 (2.02)	8.60 (2.14)	—

FF = standard F-statistic testing association between instruments and each principal component; cFF = conditional F-statistic accounting for other principal components. "—" indicates that cross-validation selected $K = 2$ components (no PC3 was retained). Values shown as mean (SD) over 500 simulation replications.

Supplementary Figures

Supplementary Figure S1

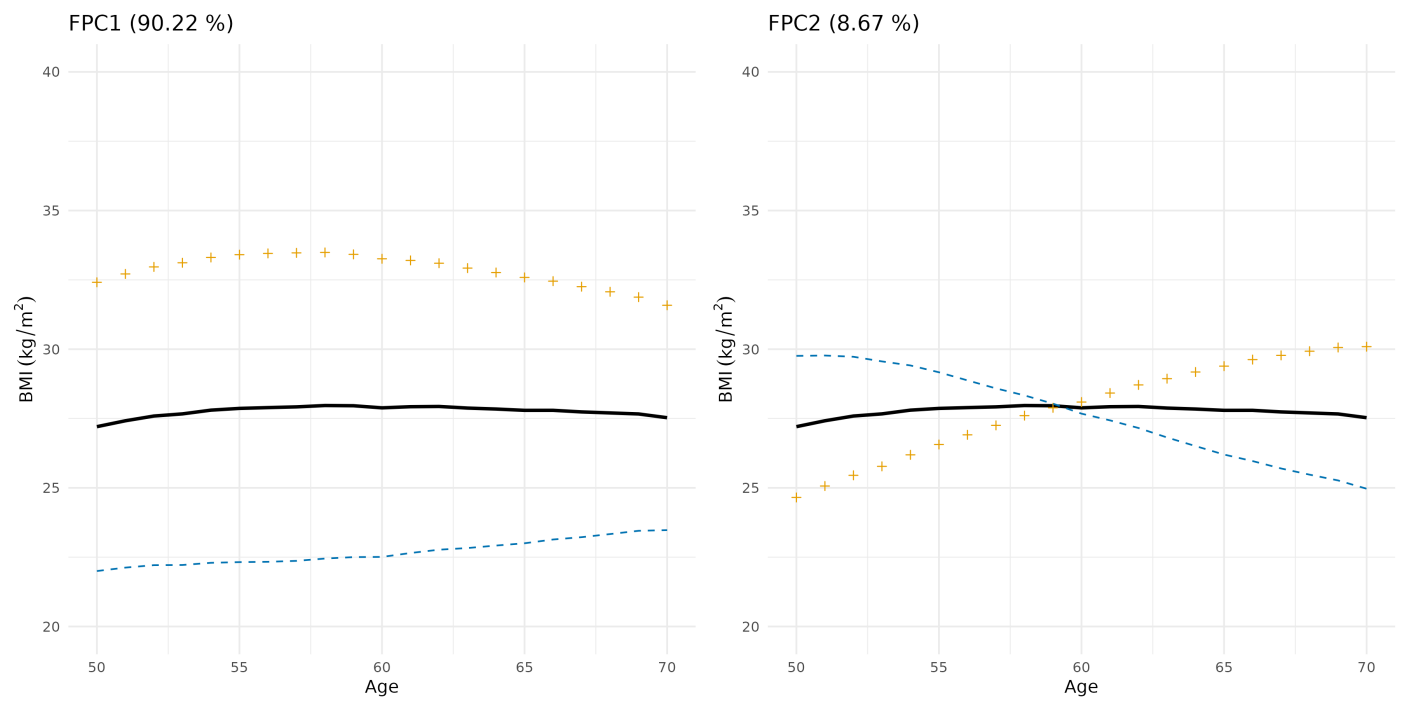

Figure S1 Functional principal component eigenfunctions for BMI trajectories in the primary analysis (T2D outcome, main cohort; $n \geq 3$ measurements). The solid black line represents the mean BMI trajectory, while the blue dashed and orange dotted lines illustrate positive and negative variation around the mean captured by each functional principal component. Left panel shows the first eigenfunction $\phi_1(t)$, right panel shows the second eigenfunction $\phi_2(t)$. Alt text: Two-panel plot of the first two functional principal components of BMI trajectories in the type 2 diabetes cohort with 3 measurements, capturing 90.60 percent and 8.3 percent of variance over ages 50 to 70.

Supplementary Figure S2

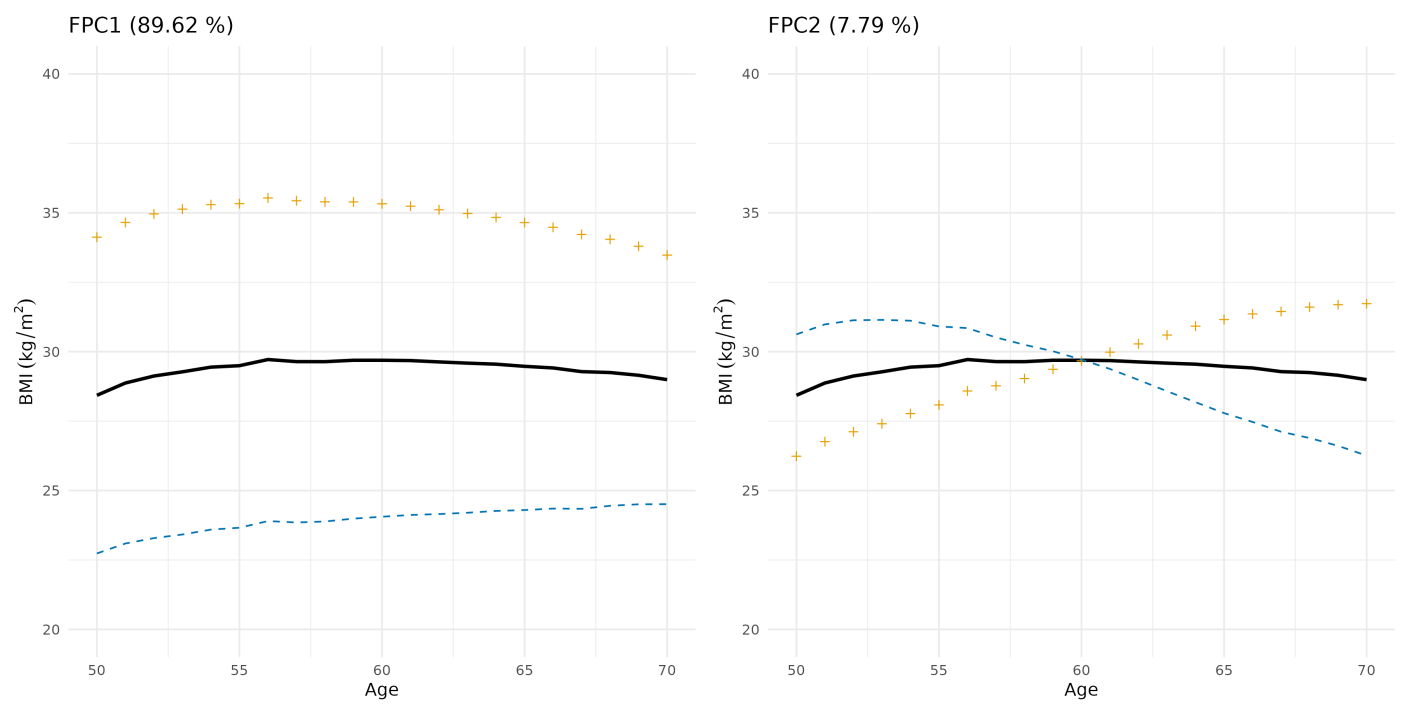

Figure S2 Functional principal component eigenfunctions for BMI trajectories in the secondary analysis (T2D outcome, sensitivity cohort; $n \geq 10$ measurements). The solid black line represents the mean BMI trajectory, while the blue dashed and orange dotted lines illustrate positive and negative variation around the mean captured by each functional principal component. Left panel shows the first eigenfunction $\phi_1(t)$, right panel shows the second eigenfunction $\phi_2(t)$. Alt text: Two-panel plot of the first two functional principal components of BMI trajectories in the type 2 diabetes cohort with 3 measurements, capturing 90.60 percent and 8.3 percent of variance over ages 50 to 70.

Supplementary Figure S3

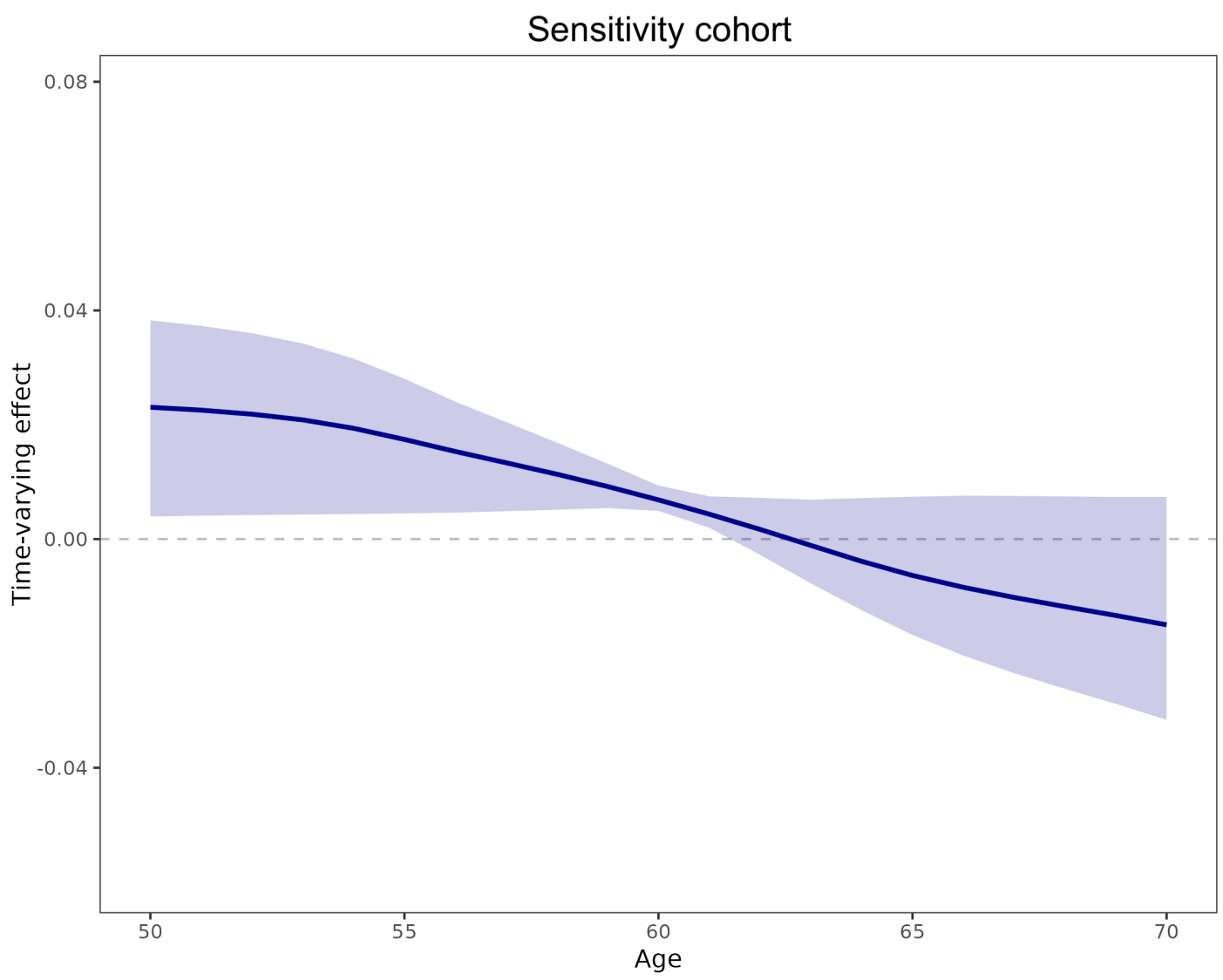

Figure S3 Genetically predicted time-varying causal effect of BMI on T2D risk in the sensitivity cohort. Blue lines represent point estimates of $\hat{\beta}(t)$ (change in log-odds of T2D per SD increase in BMI at age t), with shaded regions indicating pointwise 95% confidence intervals based on 100 bootstrap samples. The dashed horizontal line at zero indicates no effect. Alt text: Line plot of the estimated time-varying causal effect of BMI on type 2 diabetes risk in the sensitivity cohort, showing a positive effect near age 50 that declines and becomes negative after age 60, with 95 percent confidence band.

Supplementary Figure S4

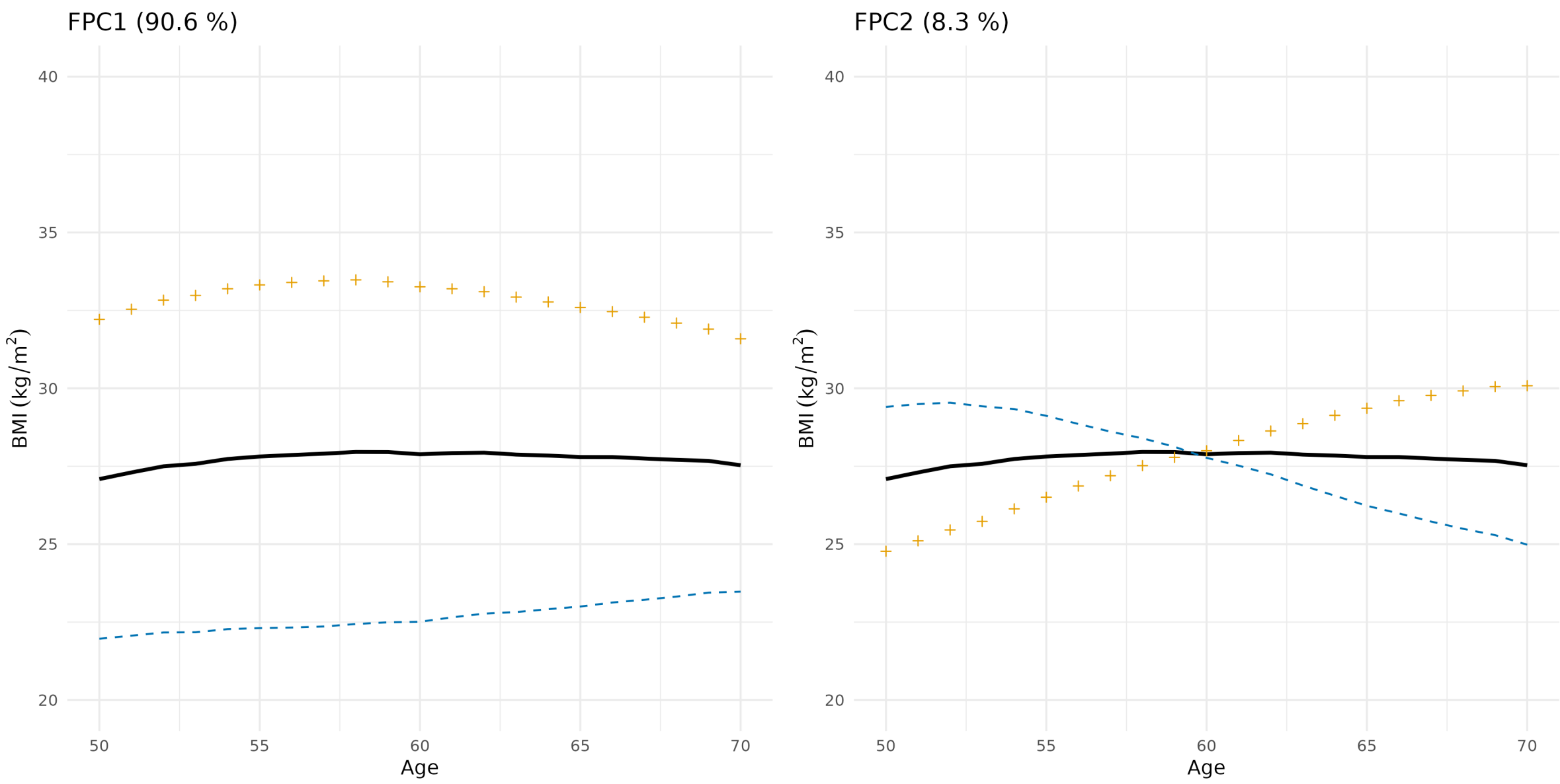

Figure S4 Functional principal component eigenfunctions for BMI trajectories in the secondary analysis (HbA1c outcome, main cohort; $n \geq 3$ measurements). The solid black line represents the mean BMI trajectory, while the blue dashed and orange dotted lines illustrate positive and negative variation around the mean captured by each functional principal component. The left panel shows the first eigenfunction $\phi_1(t)$, representing overall BMI level; the right panel shows the second eigenfunction $\phi_2(t)$, capturing linear trends over time. Alt text: Two-panel plot of the first two functional principal components of BMI trajectories in the HbA1c cohort with 3 measurements, capturing 90.60 % and 8.30 % of variance over ages 50 to 70.

Supplementary Figure S5

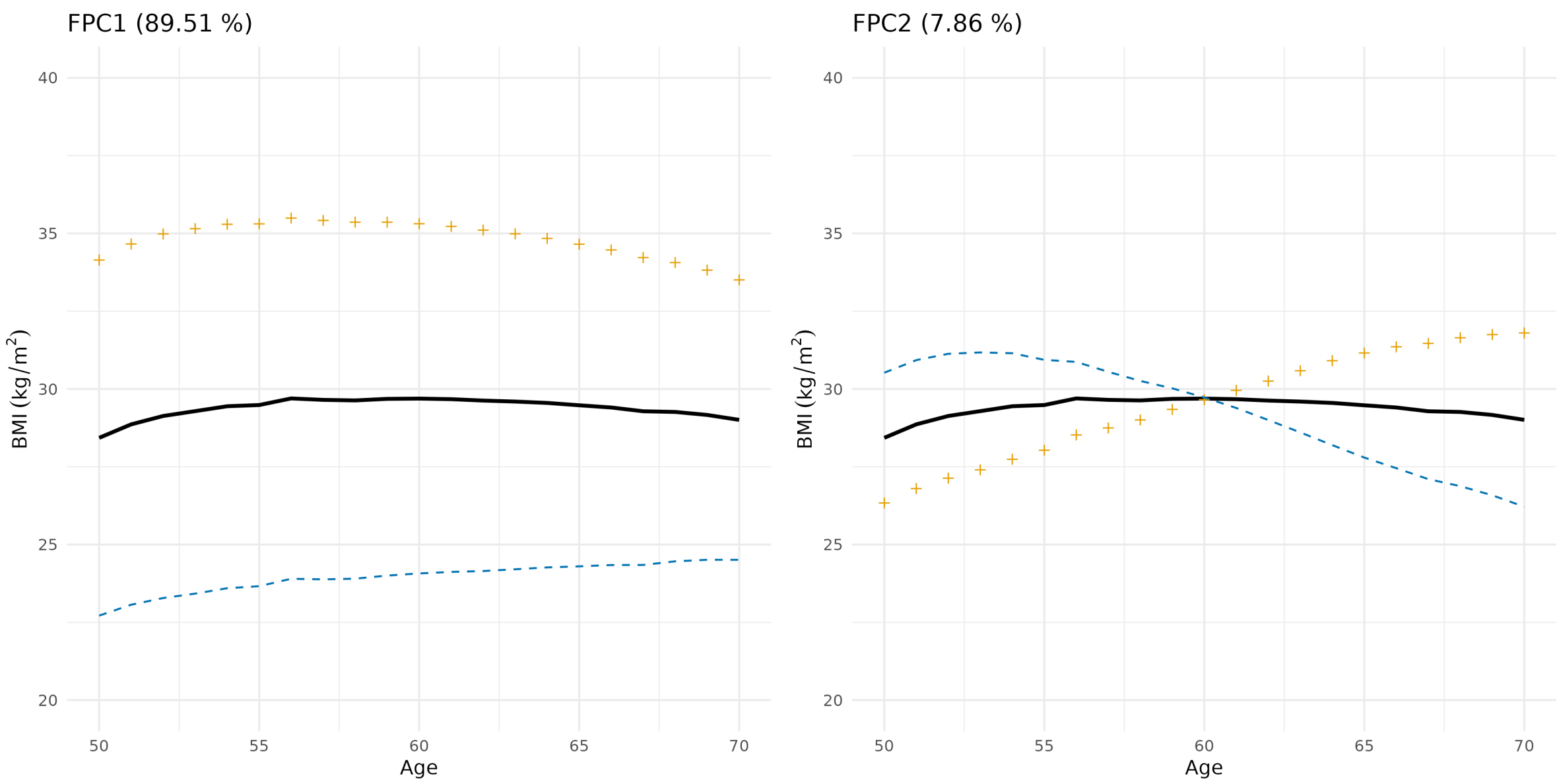

Figure S5 Functional principal component eigenfunctions for BMI trajectories in the secondary analysis (HbA1c outcome, sensitivity cohort; $n \geq 10$ measurements). The solid black line represents the mean BMI trajectory, while the blue dashed and orange dotted lines illustrate positive and negative variation around the mean captured by each functional principal component. Left panel shows the first eigenfunction $\phi_1(t)$; right panel shows the second eigenfunction $\phi_2(t)$. Alt text: Two-panel plot of the first two functional principal components of BMI trajectories in the HbA1c cohort with 10 measurements, capturing 89.51 percent and 7.86 percent of variance over ages 50 to 70.

Supplementary Figure S6

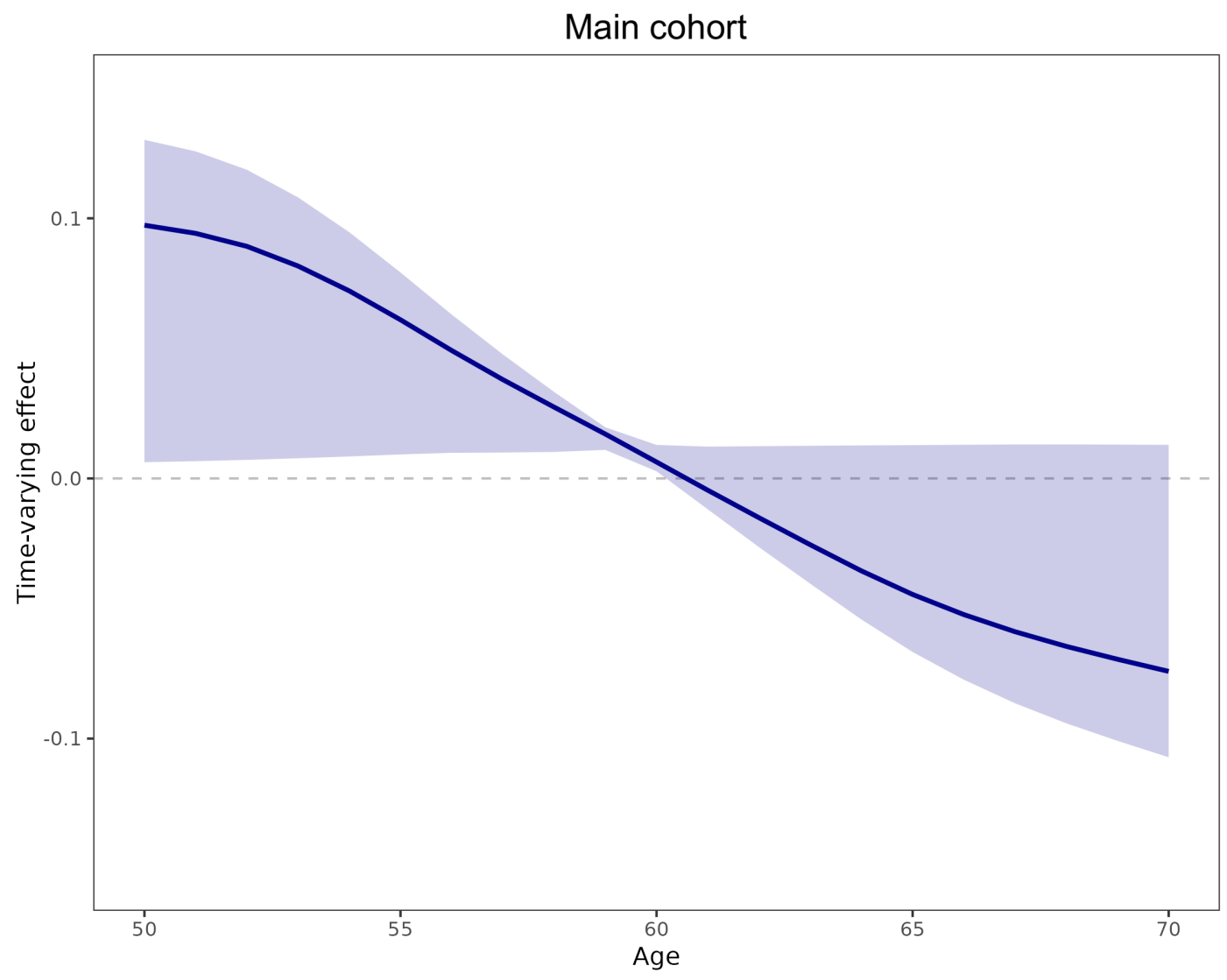

Figure S6 Genetically predicted time-varying causal effect of BMI on glycated hemoglobin (HbA1c) in the main cohort. Blue lines represent point estimates of $\hat{\beta}(t)$ (change in HbA1c [mmol/mol] per SD increase in BMI at age t), with shaded regions indicating pointwise 95% confidence intervals based on 100 bootstrap samples. Alt text: Line plot of the estimated time-varying causal effect of BMI on HbA1c levels in the main cohort, with a positive effect near age 50 that declines and becomes negative after age 60, with 95 percent confidence band.

Supplementary Figure S7

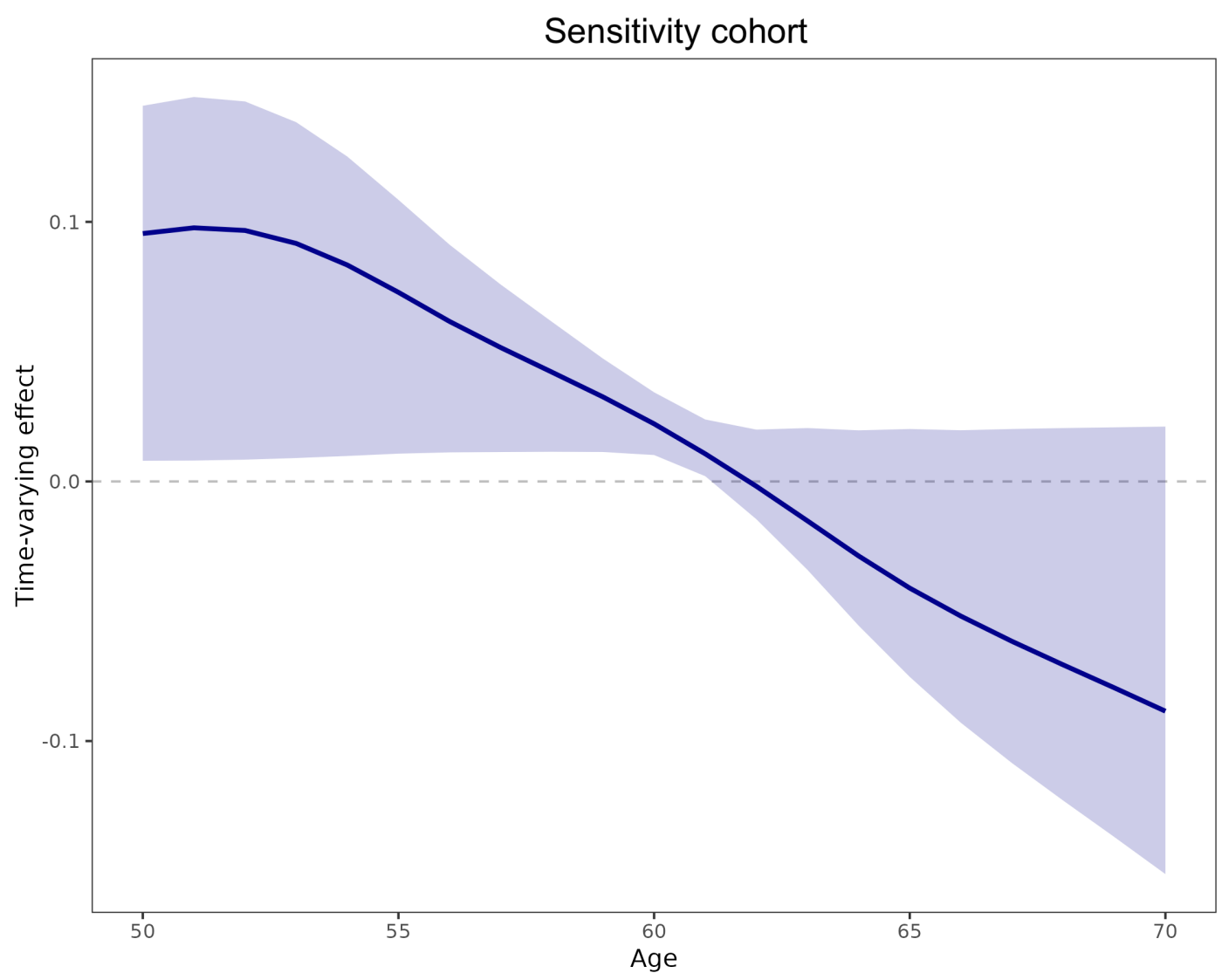

Figure S7 Genetically predicted time-varying causal effect of BMI on glycated hemoglobin (HbA1c) in the sensitivity cohort. Blue lines represent point estimates of $\hat{\beta}(t)$ (change in HbA1c [mmol/mol] per SD increase in BMI at age t), with shaded regions indicating pointwise 95% confidence intervals based on 100 bootstrap samples. Alt text: Line plot of the estimated time-varying causal effect of BMI on HbA1c levels in the sensitivity cohort, with a positive effect near age 50 that declines and becomes negative after age 60, with 95 percent confidence band.
